# Novel pain management strategy for uterine fibroid embolization

**DOI:** 10.1186/s42155-025-00516-3

**Published:** 2025-01-22

**Authors:** Elaine Ho, Kiat Tsong Tan

**Affiliations:** 1 Department of Medical Imaging, Faculty of Health Sciences, McMaster University, Hamilton, ON Canada; 2 Department of Medical Imaging, Niagara Health System, 1200 Fourth Avenue, St Catharines, ON L2S 0A9 Canada

**Keywords:** Uterine fibroid embolization, Uterine artery embolization, Fibroid, Embolization, Pain management, Nerve block, Ropivacaine

## Abstract

**Background:**

Uterine fibroid embolization can be associated with significant pain due to fibroid ischemia and interventions of the procedure itself. Fentanyl and midazolam are commonly provided for sedation and pain relief, but are not tolerated by all patients. This report outlines a novel pain management strategy for uterine fibroid embolization in a patient who could not receive either opioids or benzodiazepines.

**Methods:**

A 51 year old woman presenting with menorrhagia due to uterine fibroids was referred to interventional radiology for embolization. She was allergic to most opiates and had previously become agitated with IV midazolam, resulting in termination of a previous attempt at embolization. Thus, a combination of three analgesic modalities was used: intraarterial ropivacaine in the uterine arteries, superior hypogastric nerve block with ropivacaine, and intravenous acetaminophen. The patient underwent successful embolization and reported only intermittent pain of 1–2 out of 10 intensity.

**Discussion:**

This combined analgesic cocktail represents a novel alternative to traditional sedation for uterine fibroid embolization and may serve as a viable option for patients with similar contraindications.

## Background

Uterine fibroid embolization (UFE) is a minimally invasive technique for treating leiomyomas, performed since 1995 [1].

The mainstays of pain management for UFE are fentanyl and midazolam for sedation [2]. Post-procedural pain for UFE can be significant in patients, peaking in a few hours post procedure [2]. Various strategies have been described to manage pain post-UFE, including superior hypogastric nerve (SHN) block, intraarterial lidocaine, patient-controlled anesthesia, and non-steroidal anti-inflammatory drugs [2].

### Technique

**Fig. 1:**
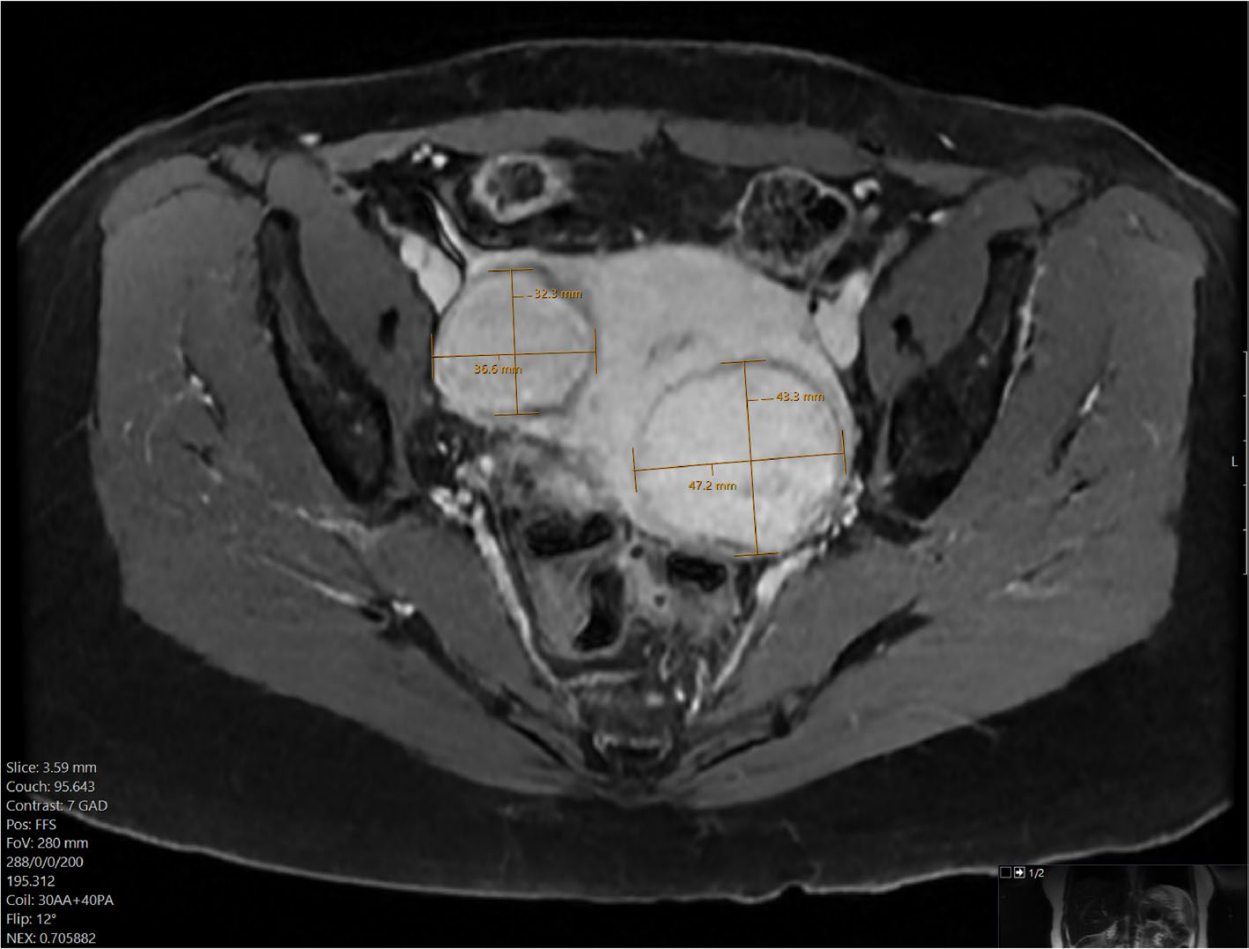
The patient’s MRI prior to embolization showed two uterine fibroids measuring 32.3 × 36.6 mm on the right and 47.2 × 43.3 mm on the left

The patient was a 51 year old 50 kg woman with mild pelvic pain and severe menorrhagia requiring blood transfusion. She was diagnosed with two uterine fibroids on ultrasound and confirmed on MRI (Fig. 1). The patient was initially treated with leuprolide acetate but developed cataracts. As she was reluctant to undergo hysterectomy, she was referred to interventional radiology for embolization. Due to her allergy to most opioids, we first attempted to undertake the procedure without fentanyl, using only midazolam for sedation. However, she had a paradoxical reaction to midazolam, becoming agitated and anxious, and the procedure was aborted. The patient was still keen to undergo UFE and wanted to try the procedure without sedation.

After an extensive literature search, we settled on a drug cocktail based on personal experience and the limited evidence available: intra-arterial ropivacaine, superior hypogastric nerve block, IV acetaminophen, and pre-procedural IV ketorolac.

The patient first received 30 mg IV ketorolac before the procedure. Then, the left uterine artery was accessed via an up-and-over technique using 5 French C2 and 2.4 French Progreat catheters. She received 1 mL 0.5% ropivacaine (5 mg/mL) intrarterially. The uterine artery was embolized using 500–700 micron beads to a pruned-tree appearance followed by another 1 mL IA 0.5% ropivacaine.

**Fig. 2:**
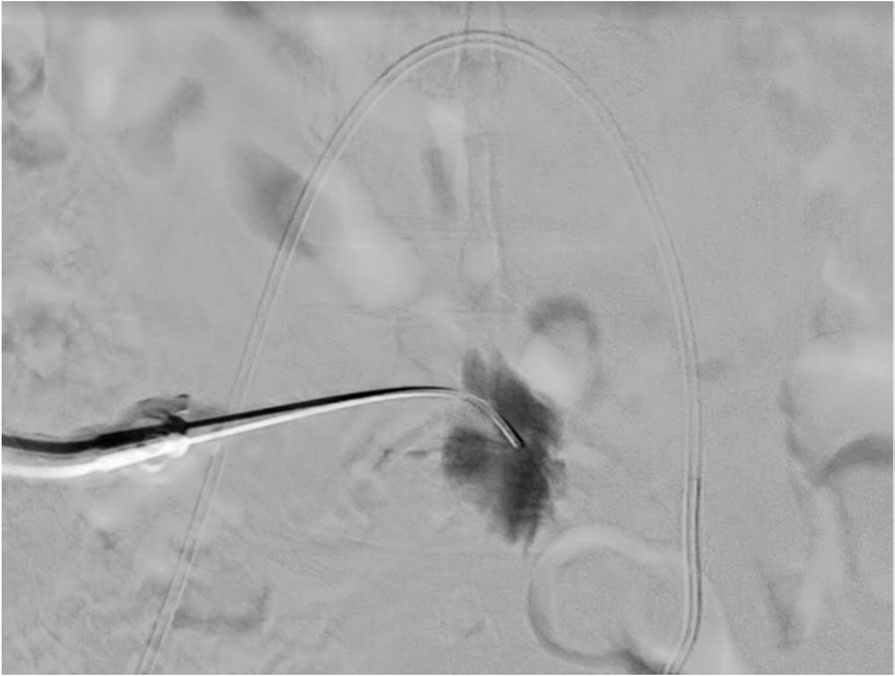
Contrast injection under fluoroscopy to confirm placement of superior hypogastric nerve block

SHN block was then performed using a 20-gauge Chiba needle inserted transabdominally at the L5 level. The placement was confirmed by contrast injection under fluoroscopy (Fig. 2). 16 mL 0.5% ropivacaine was administered.

The right uterine artery was then catheterized. A slow IV infusion of 1 g acetaminophen was commenced. 1 ml of IA 0.5% ropivacaine was administered followed by embolization using 500–700 micron beads. Once a pruned-tree appearance was achieved, a further 2 ml of 0.5% ropivacaine was given.

Cardiac and neurological monitoring was performed throughout the procedure, particularly during ropivacaine administration. There was no adverse change in either cardiac or neurological status during the procedure. The patient had no pain during and immediately after the embolization and did not require any additional analgesia in the immediate post procedural period. She was discharged four hours post-procedure at which time she reported only 1–2/10 pain on the standard Numerical Rating Scale. Discharge medications included diclofenac 50 mg three times a day and acetaminophen to take every six hours as needed. The patient did not take any of her prescribed outpatient medication as she only had mild pain post procedure.

**Fig. 3:**
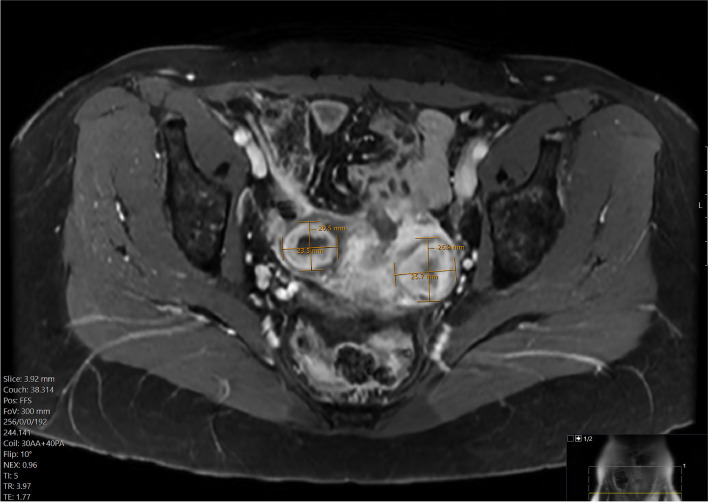
The patient’s MRI four months after embolization showed reduction in both fibroids – 23.5 × 20.5 mm on the right and 25.7 × 26.9 mm on the left

At four month follow-up, the patient’s second MRI showed successful reduction in both fibroids (Fig. 3). She also reported resolution of her symptoms.

## Discussion

This novel drug cocktail allowed a patient to undergo successful fibroid embolization despite her opioid and benzodiazepine contraindications, sparing her from the far more invasive option of hysterectomy. The individual techniques that make up this cocktail were chosen based on current literature.

SHN block has been shown to be highly effective at providing analgesia for uterine fibroid embolization. Its use is associated with significantly lower postprocedural opiate requirements [3]. This technique was also chosen due to its longer duration (8–10 h) [3], as the patient’s contraindications prevented her from receiving opioids at discharge as well. The choice to perform SHN block after the first embolization (of the left uterine artery) was so that placement of the embolization catheter could mark the aortic bifurcation, obviating the need for an angiogram and additional radiation exposure. From the author’s experience, the onset of pain usually occurs 10–30 min after embolization and peaks at 6–8 h [2], by which time the nerve block and IV acetaminophen (given intra-procedurally) would be in effect. Performing the block after the first embolization allows for a slightly longer period of analgesia post-procedure, particularly if there is difficulty in accessing the uterine artery.

Currently, there is no literature on IA ropivacaine’s use specifically in uterine fibroid embolization—as such this technique is deserving of further research on safety despite this case example. Ropivacaine, instead of lidocaine, was chosen as the intra-arterial local anaesthetic due to its longer duration of action in this patient who was intolerant of opiates. Nevertheless, the choices of agent and dose were still informed by research evidence. The main risk of any intravascular local anesthetic is cardiotoxicity and seizure [4]; hence, selection of ropivacaine was based on its lower cardiotoxic profile compared to other local anesthetics. Ropivacaine has a lower risk of arrhythmia than bupivacaine and levobupivacaine, as well as less myocardial depression risk than lidocaine [4]. While there is no literature on maximum intra-arterial dose of ropivacaine, the mean maximum tolerated intravenous dose ± 1 standard deviation is 124 ± 38 mg [5], well above the total 25 mg received by this patient. Nevertheless, lipid emulsion was on hand in the event of toxicity.

In terms of efficacy, selection of ropivacaine was derived from similar data on IA lidocaine, which has been widely used in UFE. A randomized control trial showed that IA lidocaine reduced patients’ post-UFE numerical pain rating and narcotic requirements compared to control [6].

For the embolization itself, the use of 500–700 micron beads has been shown in one study to produce lower post-procedural pain scores than 350–500 micron beads [7]. While there is no literature on this specific sequence, the decision to deliver IA ropivacaine split into two doses (pre- and post-embolization) arose from two reasons. First, any local anesthetic carries a risk of arterial spasm [4] that would rise with a single higher dose. Secondly, the aim of the first dose is to premedicate the uterine nerve endings. A very low dose of 5 mg was administered. The embolization was then performed, which would allow the detection of any adverse cardiac or neurological event. A second dose was then administered to allow for a more prolonged analgesic effect. The author KT has used intraarterial ropivacaine successfully in drug-eluting bead, irinotecan hepatic embolization (unpublished data), which is often associated with severe prolonged pain, in just over five patients. This technique is therefore deserving of further study.

Another option for analgesia which was identified from the literature was epidural placement, which has been shown to have superior effect in UFE [8]. This option was considered but ultimately discarded due to the relative invasiveness, expense, and potential complications of epidurals. Nevertheless, if the patient had experienced uncontrolled pain, epidural placement would be a good secondary option.

## Conclusion

Fentanyl and midazolam are the mainstays of procedural sedation. In this case of a patient who was unable to tolerate either, the combination of SHN block, IA ropivacaine, pre-procedural IV ketorolac and intraprocedural IV acetaminophen allowed her to successfully undergo uterine fibroid embolization with only intermittent pain of 1–2/10 intensity. The technique which most distinguishes this patient’s management is the IA ropivacaine for which there is no other mention in the literature. While very similar to IA lidocaine used in UFE, the dose and sequence of administration in this patient were unique and deserving of further study on risk and efficacy.

## Data Availability

Data sharing is not applicable to this article as no datasets were generated or analysed during the current study.

## Abbreviations

UFE: Uterine fibroid embolization
IA: Intra-arterial
SHN: Superior hypogastric nerve
IV: Intravenous

## References

[1] Young M, Coffey W, Mikhail LN. Uterine Fibroid Embolization. StatPearls, Treasure Island (FL): StatPearls Publishing; 2024. 30085558

[2] du Pisanie JL, Commander CW, Burke CT. Management of Postprocedural Uterine Artery Embolization Pain. Semin Interv Radiol. 2021;38:588–94. 10.1055/s-0041-1739161. 10.1055/s-0041-1739161 PMC8612840 34853507

[3] Park PJ, Kokabi N, Nadendla P, Lindsey T, Dariushnia SR. Efficacy of Intraprocedural Superior Hypogastric Nerve Block in Reduction of Postuterine Artery Embolization Narcotic Analgesia Use. Can Assoc Radiol J J Assoc Can Radiol. 2020;71:75–80. 10.1177/0846537119888391. 10.1177/0846537119888391 32062997

[4] Groban L, Dolinski SY. Differences in cardiac toxicity among ropivacaine, levobupivacaine, bupivacaine, and lidocaine. Tech Reg Anesth Pain Manag. 2001;5:48–55. 10.1053/trap.2001.23679.

[5] Product monograph – ropivacaine hydrochloride, USP [Internet]. Toronto (ON): Fresenius Kabi Canada Ltd.; 2018 Oct 29 [cited 2024 Dec 20]. Available from: https://pdf.hres.ca/dpd_pm/00048075.PDF.

[6] Noel-Lamy M, Tan KT, Simons ME, Sniderman KW, Mironov O, Rajan DK. Intraarterial Lidocaine for Pain Control in Uterine Artery Embolization: A Prospective, Randomized Study. J Vasc Interv Radiol JVIR. 2017;28:16–22. 10.1016/j.jvir.2016.10.001. 27884686 10.1016/j.jvir.2016.10.001

[7] Bilhim T, Pisco JM, Duarte M, Oliveira AG. Polyvinyl alcohol particle size for uterine artery embolization: a prospective randomized study of initial use of 350–500 μm particles versus initial use of 500–700 μm particles. J Vasc Interv Radiol JVIR. 2011;22:21–7. 10.1016/j.jvir.2010.09.018. 21106390 10.1016/j.jvir.2010.09.018

[8] Malouhi A, Aschenbach R, Erbe A, Owsianowski Z, Rußwurm S, Runnebaum IB, et al. Effectiveness of Superior Hypogastric Plexus Block for Pain Control Compared to Epidural Anesthesia in Women Requiring Uterine Artery Embolization for the Treatment of Uterine Fibroids - A Retrospective Evaluation. ROFO Fortschr Geb Rontgenstr Nuklearmed. 2021;193:289–97. 10.1055/a-1231-5649. 32882725 10.1055/a-1231-5649

